# A Study to Assess the Factors Affecting Adherence to Exercise in the Indian Population

**DOI:** 10.7759/cureus.6062

**Published:** 2019-11-03

**Authors:** Shruti Shettigar, Kiran Shivaraj, Sneha Shettigar

**Affiliations:** 1 Internal Medicine, Rajarshee Chhatrapati Shahu Maharaj Government Medical College and CPR Hospital, Kolhapur, IND; 2 Internal Medicine, Brookdale University Hospital and Medical Center, Brooklyn, USA; 3 Preventive Medicine, SMBT Institute of Medical Sciences & Research Centre, Igatpuri, IND

**Keywords:** exercise, physical activity, lifestyle, indian population

## Abstract

Introduction

As proven by many previous studies, physical inactivity is associated with many diseases, including heart conditions and cancer. The elimination of physical inactivity helps increase life expectancy and reduce morbidity. Nonadherence to exercise is a common problem faced by many people. The goal of this study was to determine the percentage of people in the Indian population who regularly exercise. We also assessed factors for nonadherence, motivating factors, and the intensity of exercise usually performed and explored any association between adherence to exercise and demographic factors.

Materials and methods

We conducted an anonymous questionnaire-based, cross-sectional study in an adult Indian population (participants were older than 18 years) from rural and urban areas having no contraindication to at least some form of voluntary exercise. Data were collected via email by sending a questionnaire, and an appropriate statistical methodology was used to derive the results.

Results

This study included 220 individuals older than 18 years, and most participants were aged 25 to 30 years (35.5%). Most participants (67.3%) were women, and 32.7% were men. Forty-one percent of the total population reported suffering from some medical condition. Nearly half (51.8%) of the subjects were involved in physical activity, and 48.2% were not involved in physical activity. The most common reason for not exercising was a lack of time followed by a lack of motivation. Maintenance of good health was the main reported benefit of physical exercise, with self-motivation being the main motivator. Low-intensity exercise was the preferred form of exercise for most of the participants; high-intensity exercise was preferred by younger participants, though less commonly than low-intensity exercise. Older participants (i.e., those aged > 40 years) preferred moderate-intensity exercise. Only half the study population were educated regarding physical activity by a healthcare professional. We found no statistically significant association between the presence of a medical condition, body mass index (BMI), or healthcare education and adherence to exercise.

Conclusion

The inclusion of exercise in a daily routine is one of the more important lifestyle changes advised for all ailments and for improvements in patient quality of life. Nearly half the Indian population does not exercise daily. Because we found no statistical significance between demographic factors, health conditions, BMI, or general healthcare education, an individualized education and exercise plan may help improve exercise adherence.

## Introduction

The American College of Sports Medicine recommends that adults should engage in at least 30 minutes of moderate-intensity exercise five times per week, vigorous-intensity exercise for at least 20 minutes per day, three days per week, or a combination of both moderate and vigorous activity [1]. Given the growing, aging population and unhealthy lifestyle choices, the incidence of chronic diseases has increased [2-3]. The number of patients with diabetes has quadrupled, especially in low- and middle-income countries [2]. The prevalence of hypertension in 2010 was 28.5% to 31.5%, and the age-standardized increase in prevalence was greater in low- to middle-income countries as compared to high-income countries (where the prevalence was reduced) [3]. Exercise or physical activity is beneficial in reducing cardiovascular risks in patients with chronic diseases such as diabetes and hypertension [4-5]. One study reported that the elimination of physical inactivity would increase the life expectancy of the world's population by 0.68 years (range, 0.41-0.95 years) [6].

The same study also estimated that worldwide, physical inactivity causes 6% of the burden of disease from coronary heart disease, 7% of the type 2 diabetes burden, 10% of the breast cancer burden, and 10% of the colon cancer burden. Inactivity causes 9% of premature mortality or more than 5.3 million of the 57 million deaths that occurred worldwide in 2008 [6]. If inactivity were not eliminated but decreased instead by 10% or 25%, more than 533,000 deaths and more than 1.3 million deaths, respectively, could be averted every year [6].

Typically, 50% of the individuals who begin an exercise program stop within the first six months [7]. The challenge usually faced is having the right motivation to inculcate exercise into the daily routine, finding time for it in a busy schedule, and sticking to the regimen for a long duration. The goal of this study was to determine the percentage of people in India who regularly exercise. We also analyzed factors for nonadherence to exercise regimens, motivating factors, and the intensity of exercises performed, and we explored the associations between adherence to exercise and demographic data.

## Materials and methods

We conducted an anonymous survey of adults (those aged 18 or older) in an Indian population from both urban and rural areas. The purpose of the study was explained to the participants, and a questionnaire was designed and sent to the participants via email. We included patients older than 18 years who had no contraindication to at least some form of voluntary exercise. As it was a voluntary study, we assumed that all subjects who completed and returned the questionnaire implied consent to include their responses in the study results. Those who were not able to engage in any form of exercise due to disability or medical reasons were excluded from the study. The data obtained after compiling the survey answers were analyzed for various factors affecting adherence to exercise, including demographic factors, medical conditions, and access to health care.

## Results

A total of 220 respondents (67.3% men (n=148), 32.7% women (n=72)) were included in the study. The largest age group was of respondents aged 25 to 30 years (35.5%; Table 1).

**Table 1:** Distribution of sample according to age

Age (Years)	Frequency (n)	Percent
18 – 25	63	28.6%
25 – 30	78	35.5%
30 – 40	21	9.5%
> 40	58	26.4%
Total	220	100%

Most of the respondents (n=204, 92.7%) resided in an urban area, and 16 (7.3%) resided in rural areas. Nearly half of all respondents (50.5%) had graduated from college, and 40.5% completed postgraduate education. Forty-one respondents (18.6%) reported suffering from some form of medical condition. 

Slightly more than half of the respondents (n=114, 51.8%) reported involvement in some physical activity regularly. The remaining 106 participants (48.2%) were not exercising regularly (Table 2).

**Table 2:** Exercise frequency

Assessment according to whether doing exercise on a regular basis	Frequency (n)	Percent
No	106	48.2%
Yes	114	51.8%
Total	220	100%

Most respondents (n=127, 57.7%) reported engaging in some physical exercise in the last week. Only 20 (9.1 %) respondents reported not remembering the last time they exercised (Table 3).

**Table 3:** Time since last exercise

Assessment according to when last time did exercise	Frequency (n)	Percent
Do not remember	20	9.1%
Last week	127	57.7%
>1 week	18	8.2%
>1 month	39	17.7%
>1 year	16	7.3%
Total	220	100%

**Figure 1:**
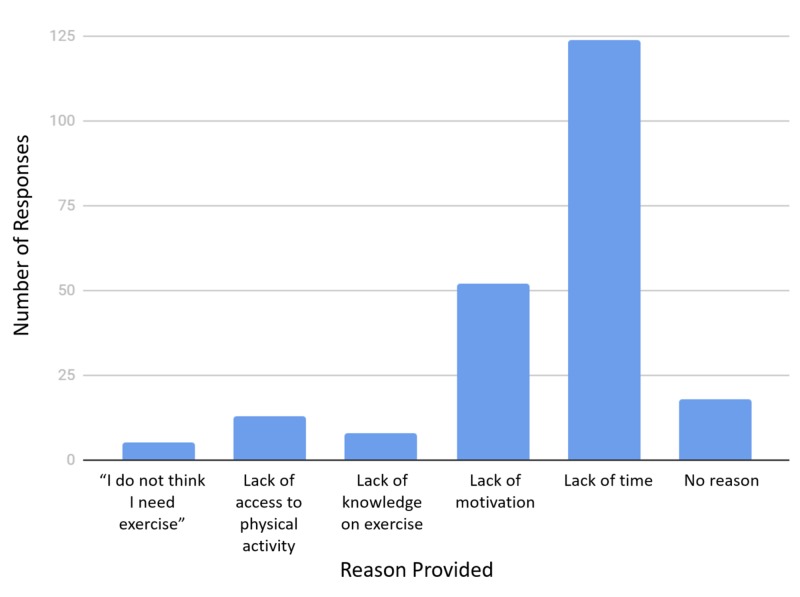
Assessment according to the main reason for not exercising

The most common reason given for not exercising was lack of time (Figure 1), reported by 56.4% of the population. Lack of motivation was the second most common reason, as seen in 23.6% of the population.

The most common reason for exercising was to maintain good health, according to 69.1% of the study population; weight loss was the given reason by only 13.2% of the study subjects.

Among 220 respondents, 101 (45.9%) respondents did not receive any advice from any healthcare professional. Ninety-two (41.8%) respondents reported receiving advice from a healthcare professional, and the remaining 27 (12.3%) respondents were not sure whether they received any advice from any healthcare professional (Table 4).

**Table 4:** Counseling from a healthcare professional regarding physical activity

Assessment according to whether received any advice from a healthcare professional regarding physical activity	Frequency (n)	Percent
Maybe	27	12.3%
No	101	45.9%
Yes	92	41.8%
Total	220	100%

Of those surveyed, 119 (54.1%) said self-motivation was their motivation for exercise. Fifty-four (24.5%) people said a health condition was the main motivation for exercising (Table 5).

**Table 5:** Exercise motivation

Motivation for exercise	Frequency (n)	Percent
Friend, family, spouse, personal trainer	41	18.6%
Health condition	54	24.5%
Self-motivation	119	54.1%
Social media	6	2.7%
Total	220	100%

Many respondents (n=97, 44.1%) preferred low-intensity exercises like slow cycling, slow walking, or yoga, and 89 (40.5%) respondents said they engaged in moderate-intensity exercise like brisk walking, jogging, or dancing. Only 34 (15.5%) respondents said they did high-intensity exercise like running, aerobic dancing, or swimming (Table 6).

**Table 6:** Exercise by intensity/type

Exercise	Frequency (n)	Percent
High-intensity (running, aerobic dancing, swimming)	34	15.5%
Low-intensity (slow cycling, slow walking, yoga)	97	44.1%
Moderate-intensity (brisk walking, jogging, dancing)	89	40.5%
Total	220	100%

We conducted a chi-square test (Table 7) to determine whether adherence to exercise is associated with selected demographic variables such as age, gender, and education. The calculated chi-square value of age, gender, and education are less than their respective chi-square table value at 0.05 levels. Thus, there is no statistically significant difference between groups of demographic variables of age, gender, and education with respect to adherence to exercise.

**Table 7:** Association of demographic with adherence to exercise Abbreviations: df, degree of freedom; X², chi-square statistic

Demographic variable		Adherence to exercise		df	Calc. X² value	Table X² value	p-value	Whether significant at 0.05 level
		No	Yes					
Age (years)	18 – 25	28	35	3	7.38	7.82	0.061	No
	25 – 30	44	34					
	30 – 40	13	8					
	>40	21	37					
Gender	Male	36	36	1	0.14	3.84	0.707	No
	Female	70	78					
Education	12th year and below	10	10	2	3.69	5.99	0.158	No
	Graduate	60	51					
	Postgraduate	36	53					

We also used the chi-squared test to determine whether adherence to exercise is associated with previous medical conditions (Table 8). The calculated chi-square value is less than the respective chi-square table value at 0.05 levels. Thus, we found no statistically significant difference between medical conditions with respect to adherence to exercise.

**Table 8:** Association of medical condition with adherence to exercise. Abbreviations: df, degree of freedom; X², chi-square statistic.

Association		Adherence to exercise		df	Calc. X² value	Table X² value	p-value	Whether significant at 0.05 level
		No	Yes					
Any medical condition	No	91	88	1	2.71	3.84	0.099	No
	Yes	15	26					

Table 9 shows the association of age with different forms of exercise, such as low-, moderate-, and high-intensity exercise. The calculated chi-square value of 18.65 is more than the chi-square table value of 12.59 at 0.05 levels. Thus, we noted a statistically significant difference between age and forms of exercise. Younger age group respondents (e.g., 18 to 25-year-olds and 25 to 30-year-olds) engaged in higher intensity exercise compared to older respondents. Thus, statistically, age is associated with different forms of exercise.

**Table 9:** Association of age with form of exercise Abbreviations: df, degree of freedom; X², chi-square statistic

Association of age with form of exercise		Form of exercise			df	Calc. X² value	Table X² value	p-value	Whether significant at 0.05 level
		Low Intensity	Moderate Intensity	High Intensity					
Age (years)	18 – 25	35	18	10	6	18.65	12.59	0.0048	Yes
	25 – 30	26	36	16					
	30 – 40	9	8	4					
	>40	19	35	4					

Table 10 shows the association of BMI with adherence to exercise. As BMI data are in parametric format, we used the unpaired ‘t’ test to find the difference in mean BMI with respect to adherence to exercise. The calculated ‘t’ test value of 0.73 is less than the ‘t’ test table value of 1.96 at 0.05 levels with a degree of freedom of 218. Thus, we found no statistically significant difference in mean BMI with respect to adherence to exercise.

**Table 10:** BMI and adherence to exercise Abbreviations: BMI, body mass index; df, degree of freedom; MD, mean of the difference score; SEMD, standard error of mean of the difference score

Association of BMI with adherence to exercise		n	Mean BMI	MD	SEMD	df	Calc. ‘t’ value	Table ‘t’ value	p-value	Whether significant at 0.05 levels
Whether exercising regularly	Yes	114	24.09	0.46	0.61	218	0.73	1.96	0.468	No
	No	106	24.55							

Table 11 shows the association of healthcare advice with adherence to exercise. Twenty-seven people said they were unsure if they received healthcare advice and were excluded from this analysis. The calculated chi-square value of 1.631 is more than the chi-square table value of 3.841 at 0.05 levels. Therefore, advice from a healthcare provider was not statistically significant.

**Table 11:** Healthcare advice and adherence to exercise Abbreviations: X², chi-square

Association of healthcare advice with adherence to exercise		Adherence to exercise		Total	Calc. X² value	Table X² value	p-value	Whether significant at 0.05 level
		Yes	No					
Healthcare advice received	Yes	54	38	92	1.631	3.841	0.2013	No
	No	50	51	101				

Age, gender, or education were not factors affecting whether a person exercises or not, and, therefore, a separate intervention targeting a specific group based on age, gender, or educational status would not be effective. The presence of a pre-existing medical condition did not influence a person towards better compliance with an exercise regime; this might be due to the low number of respondents who received education regarding the benefits of exercise. There was a significant association between age and the intensity of exercise, with more subjects in the younger population engaging in high-intensity exercise (p=0.0048), which may be attributed to the general better level of physical fitness in younger respondents.

## Discussion

The age distribution of the study population showed a relatively younger population, with 64% of the subjects between the ages of 18 and 30 years with 92.7% of them residing in an urban area - these are mainly due to the design of the study, which requires the accessibility to the Internet either on the computer or on the phone. Many of the patients were educated with either a graduate or postgraduate degree; this again can be attributable to the study population being predominantly urban where access to education is more readily available than in rural areas. About half the population had a routine habit of exercising, which closely matched with the reported data that 57.7% did some exercise in the last week. Even among the patients who did not exercise regularly, a very small proportion of about 9.1% reported they did not remember the last time they exercised, suggesting a large number of the people in the study population participated in some physical activity. Our results align with Lawton et al.’s report [8] on a south Asian population who migrated to the United Kingdom in that a lack of time was the most commonly stated reason for not exercising. A significant proportion of respondents (23.6%) also mentioned a lack of motivation as the reason for the lack of physical activity, which, according to previous studies, can be modified by health education [9], yet only 41% of our respondents recalled a health professional advising them on levels of physical activity. Surprisingly, most of the population preferred to do low- to moderate-intensity exercise, which, as per previous studies, has shown to have better compliance when compared to high-intensity exercise [9].

Despite reports that high-intensity exercise is associated with low adherence [10], a small study done by Jonathan et al. on eight healthy volunteers showed high-intensity interval running to be more enjoyable based on the physical activity enjoyment scale versus moderate-intensity continuous running [11], which might translate to better adherence to exercise in people who can participate in such an activity. There was no significant association between BMI and adherence to exercise, which is not preferable as people with higher BMI are expected to exercise regularly-which again can be attributable to lack of health education. A structured program for providing education regarding physical activity with individual-level tailoring and with population-wide initiatives may inspire better rates of participation in exercise.

Our findings indicate there is no statistically significant difference between engaging in exercise and receiving healthcare advice, which did not align with our expectations. The discrepancy may be due to the study design’s use of a questionnaire-based survey, which only assessed whether the participant received any form of advice from a healthcare professional. The survey did not involve questions regarding the level of aggressiveness or efficacy of the counseling received, whether any personalized exercise regimen was advised, or any follow-up measures were emphasized.

## Conclusions

The inclusion of exercise into the daily routine is an important lifestyle change for many patients. This anonymous questionnaire-based, cross-sectional study in an adult Indian population to assess the percentage of people in the Indian population who regularly exercise and factors for nonadherence, motivating factors, and the intensity of exercise usually performed and any association between adherence to exercise and demographic factors. Nearly half the Indian population does not exercise daily. Because we found no statistical significance between demographic factors, health conditions, BMI, or general healthcare education, we suggest measures be taken to inspire better rates of participation in exercise, perhaps via a structured program for providing tailored education regarding physical activity with individual-level tailoring and with population-wide initiatives.
